# Intelligent Energy Efficiency Maximization for Wirelessly-Powered UAV-Assisted Secure Sensor Network

**DOI:** 10.3390/s25051534

**Published:** 2025-03-01

**Authors:** Fang Xu, Xinyu Zhang

**Affiliations:** College of Electronic and Information Engineering, Southwest University, Chongqing 400715, China; zxy18982399560@email.swu.edu.cn

**Keywords:** uplink, energy efficiency, UAV, wireless powered network, downlink

## Abstract

The rapid proliferation of Internet of Things (IoT) devices and applications has led to an increasing demand for energy-efficient and secure communication in wireless sensor networks. In this article, we firstly propose an intelligent approach to maximize the energy efficiency of the UAV in a secure sensor network with wireless power transfer (WPT). All sensors harvest energy via downlink signal and use it to transmit uplink information to the UAV. To ensure secure data transmission, the UAV needs to optimize the transmission parameters to decode received information under malicious interference from an attacker. Code Division Multiple Access (CDMA) is adopted to improve uplink communication robustness. To maximize the UAV’s energy efficiency in data collection tasks, we formulate a constrained optimization problem that jointly optimizes charging power, charging duration, and data transmission duration. Applying Deep Deterministic Policy Gradient (DDPG) algorithm, we train an action policy to dynamically determine near-optimal transmission parameters in real time. Numerical results validate the superiority of proposed intelligent approach over exhaustive search and gradient ascent techniques. This work provides some important guidelines for the design of green secure wireless-powered sensor networks.

## 1. Introduction

The future Internet of Things (IoT) is anticipated to enable massive deployments of intelligent sensors that can securely and efficiently connect to the internet and interact with one another. However, powering these large-scale sensor networks sustainably remains a critical challenge, as relying on grid electricity for a vast number of devices is often impractical. Wireless power transfer (WPT) has emerged as a promising solution, allowing sensors to harvest energy wirelessly while eliminating the need for frequent battery replacements. Despite its advantages, WPT systems face significant wireless path loss and heightened security risks, making the development of secure and energy-efficient transmission schemes essential for real-world applications. Recent research has investigated the approach for optimizing secrecy performance in wireless communication networks, e.g., [1,2,3,4,5,6]. For instance, ref. [1] proposed a secure communication paradigm at physical layer for wireless-powered sensor networks, where corresponding challenges, countermeasures, and road ahead were discussed in detail. Ref. [2] proposed a certificateless linearly homomorphic signature scheme, for data transmission and authentication, to ensure the authenticity, integrity and non-repudiation of data. Ref. [3] analyzed secrecy outage probability (SOP) for dual-hop relaying systems with hybrid MIMO RF/FSO links and proposed transmit antenna selection (TAS) schemes to enhance secrecy under imperfect channel state information (CSI). Similarly, ref. [4] examined SOP for FSO systems with a distinct eavesdropper (ED) near the relay, deriving closed-form expressions for various scenarios. Additionally, ref. [5] proposed a learning-based secure transmission framework using deep neural networks to optimize transmission parameters for maximizing secrecy throughput, while [6] focused on robust secure transmission by optimizing the worst-case secrecy rate under energy-harvesting and power constraints. However, these studies primarily address secrecy issues rather than energy efficiency issues, leaving a significant gap in developing integrated frameworks that ensure energy-efficient and secure communication for intelligent sensor networks in IoT applications.

Refs. [7,8,9] further investigated the improvement approach for energy efficiency in terms of wireless communication network with eavesdroppers. Ref. [7] proposed a novel secure and energy-efficient message communication system, called MComIoV, where MComIoV was evaluated through security proof and analysis against various attacks to verify corresponding robustness. Ref. [8] proposed a beamformer design approach to optimize the energy efficiency, in terms of secrecy bits per Joule under secrecy quality-of-service (QoS) constraints, for a multi-user MIMO communication network with an eavesdropper. Ref. [9] optimized the resource allocation strategy, the UAV’s trajectory, and the jamming policy of the jammer UAV are jointly optimized for maximizing overall energy efficiency for secure unmanned aerial vehicle (UAV) communication system. However, these literature did not focus on secure communication network with wireless power transfer.

Refs. [10,11,12,13,14,15,16,17] further investigated energy efficient simultaneous information and power transfer (SWIPT) system. Ref. [10] realized high energy efficient transmission by optimizing power allocation and Energy Harvesting (EH) relay selection in clustered wireless sensor network. Ref. [11] investigated the energy efficiency maximization for coordinated multi-point (CoMP) SWIPT heterogeneous networks (HetNets) where joint beam-forming and power allocation are optimized under intra-cell and inter-cell interferences. Ref. [12] studied the optimal resource allocation and data forwarding in integrated data and energy transmission. Ref. [13] focused on the energy efficient SWIPT in MIMO system where optimal power allocation and precoding have been realized. Ref. [14] developed a tractable model in terms of joint downlink and uplink transmission of K-tier heterogeneous cellular networks by SWIPT, in which authors derive the outage probability and the average ergodic rate of a random mobile user under different cell associations. This will provide guideline for energy efficient SWIPT system. Ref. [15] proposed a heuristic algorithm to optimize the transfer time allocation based on energy consumption distribution of nodes under scene of SWIPT. Ref. [16] proposed a mode switching scheme for SWIPT on basis of random beamforming technique. This can generate artificial channel fading to increase energy harvesting efficiency of receiver. Ref. [17] has achieved the energy efficient secure transmission system by jointly optimizing energy beamformers, information beamformers and transmit time switching ratio, subject to constrained usersharvested energy as well as power budget of BS. Refs. [18,19,20,21] mainly investigated the wirelessly powered network with separate information transmission. Refs. [22,23,24] aimed at improving the energy efficiency of wireless powered network by utilizing renewable energy. Ref. [22] proposed a hybrid framework that combines the two technologies, namely solar energy harvesting and wireless charging, in which cluster head placement has been optimized to minimize corresponding energy consumption. In [23], a M/M/1 make-to-stock queuing model is proposed to investigate decentralized decisions on how much amount of renewable energy should be supplied to BS for minimizing the individual cost of renewable source. Ref. [24] investigated the tradeoff between energy consumption and QoS, where the wireless network is powered by both grid and harvested renewable energy. However, these literature did not consider the security issue and mainly use iterative algorithms to optimize transmission parameters. Since online iterative calculation needs to consume some time, these approaches can not be used for determining optimal transmission parameters under strict latency constraint.

Recently, deep reinforcement learning (DRL) become a powerful tool for handing complicated optimization issues in communication network [25]. Refs. [26,27,28] introduced the deep reinforcement learning algorithm to achieve energy efficient sensing, navigation and wireless transmission, respectively. Ref. [26] utilized distributed DDPG to enhance EE in UAV mobile sensing (MCS). Ref. [27] proposed a decentralized deep reinforcement learning (DRL) framework to arrive at energy efficient navigation for distributed UAVs. Ref. [28] proposed a reinforcement learning based energy management strategy to access good long-term average net bit rate.However, DRL approach, applicable for realizing green secure data collection in IoT, has not been investigated yet.

In a word, green secure transmission scheme for wirelessly powered sensor network was not well investigated yet in all the above literature. In this article, we design a DDPG approach to arrive at a near-optimal secure transmission scheme for maximizing the energy efficiency of data collection in wireless sensor network with malicious attackers and WPT. With well-trained policy model, optimal transmission parameters can be determined in real time. As such, our proposed approach can effectively reduce the online latency of wireless transmissions. To our best knowledge, this is the first work to investigate the adaptive scheme for green secure IoT transmissions under strict latency constraint and WPT.

## 2. System and Channel Model

**Figure 1:**
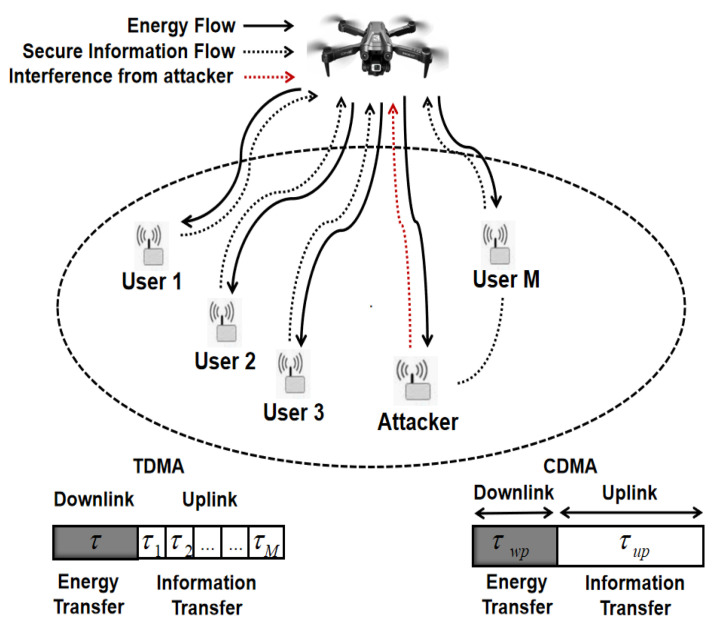
Wirelessly-powered secure sensor network using CDMA mode.

We consider a wirelessly-powered sensor network with an UAV, an attacker and multiple sensors, as illustrated in Figure 1. More specifically, the UAV firstly transfers energy to all sensors through downlink and then, all sensors send back collected data to the UAV through uplink using harvested energy. Note also that the attacker would constantly transfer interference signal to the UAV for degrading decoding performance. To achieve secure data collection, the UAV needs to be capable of normally collecting sensor data under the interference from attacker. We assume that all channels experience slow flat fading, where $g_{i}$ denotes the channel power gain from the UAV to sensor *i*, $g_{i}$ consists of fading channel gain $h_{i}$ and path loss $d_{i}^{-\alpha }$, i=1,2,3,⋯,M. As such, channel power gain is a random variable depending upon distance $d_{i}$ and path loss exponent α, i=1,2,3,⋯,M. Applying linear energy harvesting (EH) model, the harvested energy at sensor *i*, i=1,2,3,⋯,M, is given by

(1) $$E_{H_{i}}=P_{S}g_{i}\tau_{wp}\eta ,$$

where $P_{S}$ is charging power level of the UAV, $\tau_{wp}$ is the charging duration and η is DC energy transfer efficiency. All sensors’ positions are assumed to follow 2D poisson point process (PPP) with the density of λ. During uplink data collection, both TDMA and CDMA are applied to facilitate the data transmission.

### 2.1. TDMA

In terms of TDMA mode, all sensors send collected data to the UAV in turn through uplink transmission. $\tau_{i}$ is the transmission duration for sensor *i*, i=1,2,3,⋯,M. As such, effective data rate can be denoted by

(2) $$\begin{array}{c} R_{TDMA}=\sum_{i=1}^{M}\tau_{i}B\log_{2}\left(1+\frac{P_{S}g_{i}^{2}\eta \tau }{\tau_{i}\left(P_{at}g_{at}+\sigma^{2}\right)}\right)/T. \end{array}$$

Here, *B* is the effective bandwidth of wirelessly-powered network, $\sigma^{2}$ is the average noise power, *T* is the duration of data collection and $\sum_{i=1}^{M}\tau_{i}$ is equal to T−τ, $P_{at}$ is the transmit power level of the attacker and $g_{at}$ is the channel power gain from the attacker to the UAV.

### 2.2. CDMA

In terms of CDMA mode, all sensors employ orthogonal codes to simultaneously transmit collected data to the UAV. Then, while noting that CDMA can considerably reduce the inter-user interference [29], we ignore the decoding interference from all normal sensors. Accordingly, effective data rate can be denoted by

(3) $$\begin{array}{c} R_{CDMA}=\sum_{i=1}^{M}\frac{B\tau_{up}\log_{2}\left(1+\frac{P_{S}g_{i}^{2}\eta \tau_{wp}}{\tau_{up}\left(P_{at}g_{at}+\sigma^{2}\right)}\right)}{T}. \end{array}$$

Here, *B* is the effective bandwidth, $\sigma^{2}$ is the average noise power and $\tau_{up}$ is the uplink transmission duration, equal to $T-\tau_{wp}$.

## 3. Energy Efficiency Maximization

In this section, we intend to maximize the energy efficiency for wirelessly-powered sensor network under CDMA mode and TDMA mode. With TDMA mode, action policy for determining near-optimal transmission parameters is obtained, and all closed-form expressions for optimal transmission parameters are derived accordingly.

### 3.1. TDMA

#### 3.1.1. Optimization Without QoS Constraint

We maximize the energy efficiency of data collection under an attacker’s interference, which is calculated as the product of effective power and operating duration. General optimization problem is formulated as follows

$$\begin{array}{cc} \; & \max_{P_{S},\;\tau ,\;\tau_{1},\;\tau_{2},\;\cdots ,\;\tau_{M}}\;Eff=\frac{\sum_{i=1}^{M}\frac{\tau_{i}}{T}B\log_{2}\left(1+\frac{P_{S}g_{i}^{2}\eta \tau }{\tau_{i}\left(P_{at}g_{at}+\sigma^{2}\right)}\right)}{\left(P_{S}\frac{\tau }{T}+P_{C}\right)},\\ \; & \begin{array}{ccc} s.t.\; & \sum_{i=1}^{M}\tau_{i} & =T-\tau ,\;0<P_{S}\leq P_{\max}, \end{array} \end{array}$$

where $\sum_{i=1}^{M}\tau_{i}B\log_{2}\left(1+\frac{Pg_{i}^{2}\eta \tau }{\tau_{i}\left(P_{at}g_{at}+\sigma^{2}\right)}\right)/T$ is the effective throughput and $P_{S}\tau /T+P_{C}$ is the effective transmit power of the UAV. Note that iterative algorithms have high online calculative complexity and as such, they are not suitable for adaptive transmission subject to strict latency constraint. Accordingly, we intend to derive closed-form analytical expressions for optimal transmission parameters, where corresponding parameters can be determined in real time without involving any iterative calculation. First of all, we present a lemma to show the condition of energy efficiency maximization.  

**Lemma** **1.** *Optimal $\tau_{1}$, $\tau_{2}$, ⋯, $\tau_{M}$ need to satisfy the condition $P_{S}g_{1}^{2}\eta \tau /\tau_{1}=P_{S}g_{2}^{2}\eta \tau /\tau_{2}=\cdots =P_{S}g_{M}^{2}\eta \tau /\tau_{M}=C$.*

We provide corresponding proof as below

**Proof.**  We assume that $P_{S}$ and τ are given and as such, optimization problem can be updated to

$$\begin{array}{cc} \; & \max_{\tau_{1},\;\tau_{2},\;\cdots ,\;\tau_{M}}\;E_{Eff}=\frac{\sum_{i=1}^{M}\tau_{i}B\log_{2}\left(1+P_{S}g_{i}^{2}\eta \tau /\tau_{i}\left(P_{at}g_{at}+\sigma^{2}\right)\right)}{P_{S}\tau +P_{C}T},\\ \; & \begin{array}{ccc} s.t.\; & \sum_{i=1}^{M}\tau_{i} & =T-\tau . \end{array} \end{array}$$

Note that the second derivative, for this objective function, with respect to $\tau_{i}$ is always less than zero, i=1,2,⋯,M. Hence, the above function and constraint are both jointly concave with respect to $\tau_{1}$, $\tau_{2}$, ⋯ and $\tau_{M}$. According to Ref. [30], we apply KKT condition to arrive at the mathematical expression $L=EE+\lambda (T-\tau -\sum_{i=1}^{M}\tau_{i})$. As such, we can further transform $\nabla_{\tau_{1},\;\cdots ,\;\tau_{M}}L=0$ to

(4) $$\begin{array}{c} \frac{B(\ln\left(1+\frac{P_{S}g_{i}^{2}\eta \tau }{\tau_{i}\left(P_{at}g_{at}+\sigma^{2}\right)}\right)-\frac{P_{S}g_{i}^{2}\eta \tau /\left(\tau_{i}\left(P_{at}g_{at}+\sigma^{2}\right)\right)}{\left(1+P_{S}g_{i}^{2}\eta \tau /\left(\tau_{i}\left(P_{at}g_{at}+\sigma^{2}\right)\right)\right)})}{(P_{S}\tau +P_{C}T)\ln2}-\lambda =0, \end{array}$$

where i=1,⋯,M. We can see that Equation (4) can hold if and only if $P_{S}g_{1}^{2}\eta \tau /\tau_{1}=P_{S}g_{2}^{2}\eta \tau /\tau_{2}=\cdots =P_{S}g_{M}^{2}\eta \tau /\tau_{M}=C$, where *C* is a constant.

Accordingly, by substituting the condition of Lemma 1 into original optimization problem, we can rewrite corresponding problem to

$$\begin{array}{cc} \; & \max_{P_{S},\;\tau ,\;\tau_{1},\;\cdots ,\;\tau_{M}}\;E_{Eff}=\frac{B\left(\sum_{i=1}^{M}\tau_{i}\right)\log_{2}\left(1+\frac{C}{\left(P_{at}g_{at}+\sigma^{2}\right)}\right)}{P_{S}\tau +P_{C}T},\\ \; & \begin{array}{ccc} s.t.\; & \sum_{i=1}^{M}\tau_{i} & =T-\tau ,\;0<P_{S}\leq P_{\max},\\ \; & P_{S}g_{1}^{2}\eta \tau /\tau_{1} & =\cdots =P_{S}g_{M}^{2}\eta \tau /\tau_{M}=C. \end{array} \end{array}$$

According to $\sum_{i=1}^{M}\tau_{i}$=T−τ and $\frac{P_{S}g_{1}^{2}\eta \tau }{\tau_{1}}=\cdots =\frac{P_{S}g_{M}^{2}\eta \tau }{\tau_{M}}=C$, we have $C=\sum_{i=1}^{M}\frac{P_{S}g_{i}^{2}\eta \tau }{T-\tau }$ and $\tau_{i}=\left(T-\tau \right)\frac{g_{i}^{2}}{\sum_{i=1}^{M}g_{i}^{2}}$, i=1,2,⋯,M. Then, we further simplify the optimization problem to

$$\begin{array}{cc} \; & \max_{P_{S},\;\tau }\;E_{Eff}=\frac{B\left(T-\tau \right)\log_{2}\left(1+\frac{P_{S}\left(\sum_{i=1}^{M}g_{i}^{2}\right)\eta \tau }{\left(T-\tau \right)\left(P_{at}g_{at}+\sigma^{2}\right)}\right)}{\left(P_{S}\tau +P_{C}T\right)},\\ \; & \begin{array}{ccc} s.t.\; & \tau & >0,\;0<P_{S}\leq P_{\max}. \end{array} \end{array}$$

Such objective function is still not jointly concave with respect to $P_{S}$ and τ. As such, we define a variable *x* as $P_{S}\left(\tau /\left(T-\tau \right)\right)$. Then, this optimization problem can be rewritten to

$$\begin{array}{cc} \; & \max_{P_{S},\;x}\;E_{Eff}=\frac{B\log_{2}\left(1+x\left(\sum_{i=1}^{M}g_{i}^{2}\right)\eta /\left(P_{at}g_{at}+\sigma^{2}\right)\right)}{\left(x+P_{C}\left(1+\frac{x}{P_{S}}\right)\right)},\\ \; & \begin{array}{ccc} s.t.\; & x & >0,\;0<P_{S}\leq P_{\max}. \end{array} \end{array}$$

While noting that τ/(T−τ) ranges from 0 to *∞*, *x* and $P_{S}$ are independent with each other. This is because that *x* is possibly to be any value regardless of how much is $P_{S}$. Accordingly, the objective function is monotonically increasing with $P_{S}$, based on which optimal $P_{S}$ is equal to its peak value $P_{\max}$. Subsequently, such optimization problem can be transformed to an univariate optimization problem, shown as follows

$$\begin{array}{cc} \; & \max_{x}\;E_{Eff}=\frac{B\log_{2}\left(1+x\left(\sum_{i=1}^{M}g_{i}^{2}\right)\eta /\left(P_{at}g_{at}+\sigma^{2}\right)\right)}{\left(x\left(1+\frac{P_{C}}{P_{\max}}\right)+P_{C}\right)},\\ \; & \begin{array}{ccc} s.t.\; & x & >0. \end{array} \end{array}$$

By setting ∂Eff/∂x=0, we arrive at the optimal solution as below

(5) $$\begin{array}{c} x^{*}=\frac{\frac{P_{C}}{1+\frac{P_{C}}{P_{\max}}}-\frac{\left(P_{at}g_{at}+\sigma^{2}\right)}{(\sum_{i=1}^{M}g_{i}^{2})\eta }}{W_{0}\left[e^{-1}\left(\frac{P_{C}}{1+\frac{P_{C}}{P_{\max}}}\left(\frac{\sum_{i=1}^{M}g_{i}^{2}\eta }{\left(P_{at}g_{at}+\sigma^{2}\right)}\right)-1\right)\right]}-\frac{\left(P_{at}g_{at}+\sigma^{2}\right)}{(\sum_{i=1}^{M}g_{i}^{2})\eta }, \end{array}$$

where $W_{0}\left[.\right]$ denotes the positive branch of Lambert W function [31]. Accordingly, optimal charging duration τ can be calculated by

(6) $$\begin{array}{c} \tau^{*}=T-\frac{T}{1+x^{*}/P_{\max}}. \end{array}$$

Accordingly, on the basis of the derived condition $\tau_{i}=\left(T-\tau \right)g_{i}^{2}/\sum_{i=1}^{M}h_{i}^{2}$, optimal data transmission duration $\tau_{i}$, i=1,2,⋯,M, can be denoted by

(7) $$\begin{array}{c} \tau_{i}^{*}=\left(T-\tau^{*}\right)\frac{g_{i}^{2}}{\sum_{i=1}^{M}g_{i}^{2}}. \end{array}$$

#### 3.1.2. Optimization with QoS Constraint

In real application scenarios, we usually need to ensure that effective throughput is greater than a threshold for satisfying the QoS requirement. Then, updated optimization problem can be shown as follows

$$\begin{array}{cc} \; & \max_{P_{S},\;\tau }\;E_{Eff}=\frac{B\left(T-\tau \right)\log_{2}\left(1+\frac{P_{S}\left(\sum_{i=1}^{M}g_{i}^{2}\right)\eta \tau }{\left(T-\tau \right)\left(P_{at}g_{at}+\sigma^{2}\right)}\right)}{\left(P_{S}\tau +P_{C}T\right)},\\ \; & \begin{array}{ccc} s.t.\; & \tau & >0,\;0<P_{S}\leq P_{\max},\\ \; & B & \frac{\left(T-\tau \right)}{T}\log_{2}\left(1+\frac{P_{S}\left(\sum_{i=1}^{M}g_{i}^{2}\right)\eta \tau }{\left(T-\tau \right)\left(P_{at}g_{at}+\sigma^{2}\right)}\right)\geq R_{\min}, \end{array} \end{array}$$

where $R_{\min}$ is the lower bound of effective throughput. Following a similar process in Section 3.1, optimal $P_{S}$ is equal to $P_{\max}$. Accordingly, the above constraint for effective throughput can be transformed to $\frac{B(T-\tau )}{T}\log_{2}\left(1+\frac{P_{\max}\left(\sum_{i=1}^{M}g_{i}^{2}\right)\eta \tau }{\left(T-\tau \right)\left(P_{at}g_{at}+\sigma^{2}\right)}\right)\geq R_{\min}$. Through some mathematical manipulations, effective throughput constraint can be further updated to

(8) $$\begin{array}{cc} \; & -\frac{B}{R_{\min}\ln2}(W_{0}[-\frac{R_{\min}\left(P_{at}g_{at}+\sigma^{2}\right)\ln2}{BP_{\max}\left(\sum_{i=1}^{M}g_{i}^{2}\right)\eta }\exp(\frac{R_{\min}\ln2}{B}\\ \; & \left(1-\frac{\left(P_{at}g_{at}+\sigma^{2}\right)}{P_{\max}\left(\sum_{i=1}^{M}g_{i}^{2}\right)\eta }\right))]+\frac{R_{\min}\left(P_{at}g_{at}+\sigma^{2}\right)\ln2}{P_{\max}B\eta \left(\sum_{i=1}^{M}g_{i}^{2}\right)})\\ \; & \leq \frac{\tau }{T-\tau }\leq -\frac{B}{R_{\min}\ln2}(W_{-1}[-\frac{R_{\min}\left(P_{at}g_{at}+\sigma^{2}\right)\ln2}{BP_{\max}\left(\sum_{i=1}^{M}g_{i}^{2}\right)\eta }\\ \; & \exp\left(\frac{R_{\min}\ln2}{B}\left(1-\frac{\left(P_{at}g_{at}+\sigma^{2}\right)}{P_{\max}\left(\sum_{i=1}^{M}g_{i}^{2}\right)\eta }\right)\right)]+\frac{R_{\min}\left(P_{at}g_{at}+\sigma^{2}\right)\ln2}{P_{\max}B\eta \left(\sum_{i=1}^{M}g_{i}^{2}\right)}). \end{array}$$

We present corresponding proof as follows

**Proof.**  First of all, we use simple mathematical manipulations to transform effective throughput constraint to

(9) $$\begin{array}{c} \frac{-R_{\min}\ln2}{B}\left(\frac{\tau }{T-\tau }+\frac{\left(P_{at}g_{at}+\sigma^{2}\right)}{P_{\max}\left(\sum_{i=1}^{M}g_{i}^{2}\right)\eta }\right)\exp\left(\frac{-R_{\min}\ln2}{B}\left(\frac{\tau }{T-\tau }+\frac{\left(P_{at}g_{at}+\sigma^{2}\right)}{P_{\max}\left(\sum_{i=1}^{M}g_{i}^{2}\right)\eta }\right)\right)\\ \leq \frac{-R_{\min}\left(P_{at}g_{at}+\sigma^{2}\right)\ln2}{BP_{\max}\left(\sum_{i=1}^{M}g_{i}^{2}\right)\eta }\exp\left(-\frac{R_{\min}\ln2}{B}\left(\frac{\left(P_{at}g_{at}+\sigma^{2}\right)}{P_{\max}\left(\sum_{i=1}^{M}g_{i}^{2}\right)\eta }-1\right)\right). \end{array}$$

using the property of lambert W function, Equation (8) can be derived.

Also note that $\frac{\tau }{T-\tau }$ is equal to or greater than zero and as such, $-\frac{B}{R_{\min}\ln2}\left(W_{-1}\left[-\frac{R_{\min}\left(P_{at}g_{at}+\sigma^{2}\right)\ln2}{BP_{\max}\left(\sum_{i=1}^{M}g_{i}^{2}\right)\eta }\exp\left(\frac{R_{\min}\ln2}{B}\left(1-\frac{\left(P_{at}g_{at}+\sigma^{2}\right)}{P_{\max}\left(\sum_{i=1}^{M}g_{i}^{2}\right)\eta }\right)\right)\right]+\frac{R_{\min}\left(P_{at}g_{at}+\sigma^{2}\right)\ln2}{P_{\max}B\eta \left(\sum_{i=1}^{M}g_{i}^{2}\right)}\right)$ has to be greater than zero constantly for ensuring the existence of a feasible solution. Accordingly, we have $W_{-1}\left[-\frac{R_{\min}\left(P_{at}g_{at}+\sigma^{2}\right)\ln2}{BP_{\max}\left(\sum_{i=1}^{M}g_{i}^{2}\right)\eta }\exp\left(\frac{R_{\min}\ln2}{B}\left(1-\frac{\left(P_{at}g_{at}+\sigma^{2}\right)}{P_{\max}\left(\sum_{i=1}^{M}g_{i}^{2}\right)\eta }\right)\right)\right]<-\frac{R_{\min}\left(P_{at}g_{at}+\sigma^{2}\right)\ln2}{P_{\max}B\eta \left(\sum_{i=1}^{M}g_{i}^{2}\right)}$. When

$-\frac{R_{\min}\left(P_{at}g_{at}+\sigma^{2}\right)\ln2}{P_{\max}B\eta \left(\sum_{i=1}^{M}g_{i}^{2}\right)}$≤−1, this inequality is equivalent to $\frac{R_{\min}\ln2}{B}\left(1-\frac{\left(P_{at}g_{at}+\sigma^{2}\right)}{P_{\max}\left(\sum_{i=1}^{M}g_{i}^{2}\right)\eta }\right)$<$-\frac{R_{\min}\left(P_{at}g_{at}+\sigma^{2}\right)\ln2}{P_{\max}B\eta \left(\sum_{i=1}^{M}g_{i}^{2}\right)}$, since $W_{-1}\left[x\right]$ is a monotonically decreasing function. While noting that $\frac{R_{\min}\ln2}{B}$ is greater than zero, $\frac{R_{\min}\ln2}{B}\left(1-\frac{\left(P_{at}g_{at}+\sigma^{2}\right)}{P_{\max}\left(\sum_{i=1}^{M}g_{i}^{2}\right)\eta }\right)$<$-\frac{R_{\min}\left(P_{at}g_{at}+\sigma^{2}\right)\ln2}{P_{\max}B\eta \left(\sum_{i=1}^{M}g_{i}^{2}\right)}$ is not possibly to hold. When $-\frac{R_{\min}\left(P_{at}g_{at}+\sigma^{2}\right)\ln2}{P_{\max}B\eta \left(\sum_{i=1}^{M}g_{i}^{2}\right)}$>−1, $-\frac{B}{R_{\min}\ln2}\left(W_{-1}\left[-\frac{R_{\min}\left(P_{at}g_{at}+\sigma^{2}\right)\ln2}{BP_{\max}\left(\sum_{i=1}^{M}g_{i}^{2}\right)\eta }\exp\left(\frac{R_{\min}\ln2}{B}\left(1-\frac{\left(P_{at}g_{at}+\sigma^{2}\right)}{P_{\max}\left(\sum_{i=1}^{M}g_{i}^{2}\right)\eta }\right)\right)\right]+\frac{R_{\min}\left(P_{at}g_{at}+\sigma^{2}\right)\ln2}{P_{\max}B\eta \left(\sum_{i=1}^{M}g_{i}^{2}\right)}\right)$ must be greater than zero because $W_{-1}\left[-\frac{R_{\min}\left(P_{at}g_{at}+\sigma^{2}\right)\ln2}{BP_{\max}\left(\sum_{i=1}^{M}g_{i}^{2}\right)\eta }\exp\left(\frac{R_{\min}\ln2}{B}\left(1-\frac{\left(P_{at}g_{at}+\sigma^{2}\right)}{P_{\max}\left(\sum_{i=1}^{M}g_{i}^{2}\right)\eta }\right)\right)\right]$ is less than −1. Accordingly, $-\frac{R_{\min}\left(P_{at}g_{at}+\sigma^{2}\right)\ln2}{P_{\max}B\eta \left(\sum_{i=1}^{M}g_{i}^{2}\right)}$ need to be greater than −1 to satisfy minimum effective throughput constraint, which means $P_{\max}\left(\sum_{i=1}^{M}g_{i}^{2}\right)$≥$\frac{R_{\min}\left(P_{at}g_{at}+\sigma^{2}\right)\ln2}{B\eta }$. Meanwhile, $\sum_{i=1}^{M}g_{i}^{2}$ is a random variable, which may approach zero. So $P_{\max}$ has to be large enough to make such condition satisfiable. Combining Equation (6) and derivation process in III. A, optimal charging duration τ should approach zero and as such, effective throughput actually approaches $B\log_{2}\left(1+x\left(\sum_{i=1}^{M}g_{i}^{2}\right)\eta /\left(P_{at}g_{at}+\sigma^{2}\right)\right)$. Here, *x* was defined in III. A. Accordingly, minimum effective throughput constraint can be equivalent to condition that effective received signal to noise ratio (SNR) is greater than $2^{\frac{R_{\min}}{B}}-1$, denoted by $\gamma (R_{\min})$. According to the above analysis, With optimal parameters, the UAV’s received SNR is equal to $x\left(\sum_{i=1}^{M}g_{i}^{2}\right)\eta \left(P_{at}g_{at}+\sigma^{2}\right)$. Accordingly, *x* needs to be not less than $\frac{\gamma (R_{\min})}{\left(\sum_{i=1}^{M}g_{i}^{2}\right)\eta /\left(P_{at}g_{at}+\sigma^{2}\right)}$. Then, we can rewrite the optimal *x* to

(10) $$\begin{array}{c} \bar{x^{*}}=\max\left\{x^{*},\;\frac{\gamma (R_{\min})}{\left(\sum_{i=1}^{M}g_{i}^{2}\right)\eta /\left(P_{at}g_{at}+\sigma^{2}\right)}\right\}. \end{array}$$

On the basis of Equation (7), the optimal $\tau_{1}$, $\tau_{2}$, ⋯, $\tau_{M}$ can be updated accordingly.

### 3.2. CDMA

In terms of CDMA mode, to maximize overall energy efficiency of the UAV, we formulate an optimization problem as follows

$$\begin{array}{cc} \; & \max_{P_{S},\;\tau_{wp}}\;Eff=\frac{\sum_{i=1}^{M}B\left(T-\tau_{wp}\right)\log_{2}\left(1+\frac{P_{S}\eta g_{i}^{2}\tau_{wp}}{\left(P_{at}g_{at}+\sigma^{2}\right)\left(T-\tau_{wp}\right)}\right)}{P_{S}\tau_{wp}+P_{C}T},\\ \; & \begin{array}{ccc} s.t.\; & 0<P_{S}\leq P_{\max},\;0 & \leq \tau_{wp}\leq T. \end{array} \end{array}$$

We can see that it is not possibly to derive closed-form optimal solution in terms of this case. As mentioned above, iterative algorithms have high online computational complexity and can not determine near-optimal transmission parameters in real time, e.g., [7,8,9,18,19,20,21]. While noting that machine learning can well train a DNN model for performing adaptive optimal data collection, it can offer lower latency than conventional iterative algorithms. As such, we intend to design a DDPG approach to arrive at a near-optimal solution.

#### DDPG Solution

**Figure 2:**
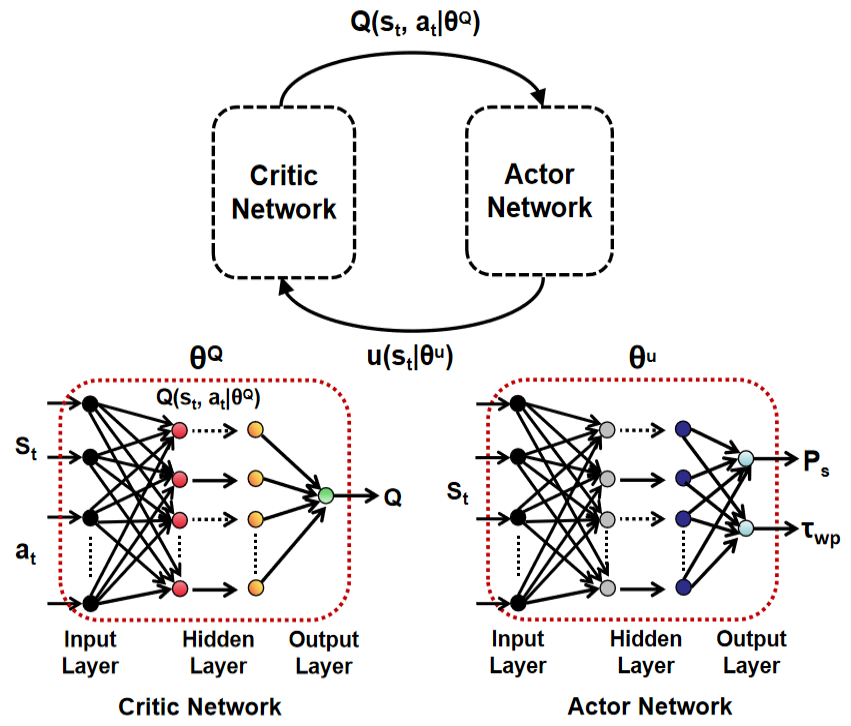
Critic and Actor network in DDPG algorithm.

Applying DDPG approach, we construct one critic network and one actor network, respectively, as illustrated in Figure 2, where $\theta^{Q}$ denotes the parameter set of critic network and $\theta^{\mu }$ denotes the parameter set of actor network. The input of critic network is the state vector and action vector, and the output is estimated *Q* value. The input of actor network is the state vector, and corresponding output is the action vector. During the training process, the critic network feeds back an estimated *Q* value for current action to the actor network to update corresponding parameter set using chain rule based gradient ascent. Adopted critic-actor configuration is presented in Figure 2, where ${\vec{s}}_{t}$ is the state vector at time instant *t* and ${\vec{a}}_{t}$ is the action vector at time instant *t*. In this case, we define the state vector ${\vec{s}}_{t}$ as ${\left[h_{1},\;h_{2},\;\cdots ,\;h_{M}\right]}^{T}$, which consists of channel gains from all sensors to the UAV at time instant *t*. Action vector ${\vec{a}}_{t}$ is defined as ${\left[P_{S},\;\tau_{wp}\right]}^{T}$. Following DDPG approach, we use expected reward function to evaluate the action policy [27], shown as follows

(11) $$\begin{array}{c} y_{t}=R_{t}\left(s_{t},a_{t}\right)+\gamma Q\left(s_{t+1},\;\mu \left(s_{t+1}|\theta^{\mu }\right)|\theta^{\mu }\right)\left|\theta^{Q}\right). \end{array}$$

Here, $y_{t}$ is the expected reward for adopted action ${\vec{a}}_{t}$, γ is the discounted factor, $R_{t}\left({\vec{s}}_{t},{\vec{a}}_{t}\right)$ is the instantaneous reward with given state ${\vec{s}}_{t}$ and action ${\vec{a}}_{t}$, ${\vec{s}}_{t+1}$ is the state at time instant t+1, $\mu ({\vec{s}}_{t+1}|\theta^{\mu })$ is the output of actor network. Prior to offline training, state transition tuples $\left\{{\vec{s}}_{i},\;{\vec{a}}_{i},\;R_{i},\;{\vec{s}}_{i+1}\right\}$, i=1,2,⋯,N, need to be randomly generated at first, and then put into a memory buffer of size *K*. Note that expected reward can be estimated using a critic network. During each training iteration, we need to obtain an action vector from current actor network and then, calculate the resulting reward as well as the state vector in the next time instant. Subsequently, one new experience tuple can be obtained for randomly replacing one existing experience tuple in the memory buffer. Note that within each training iteration, corresponding action vector is the output with random exploration, shown as below

(12) $$\begin{array}{c} {\vec{a}}_{t}=\mu \left({\vec{s}}_{t}|\theta^{\mu^{\prime }}\right)+N_{t}. \end{array}$$

$N_{t}$ denotes the variable following normal distribution with the mean value of zero and the variance of *v*. At the end of each training iteration, *v* is updated to β*v*, where β is a constant ranging from 0 to 1. After that, we can obtain the critic network parameter $\theta^{Q^{\prime }}$ through minimizing loss function, shown as follows

(13) $$\begin{array}{c} \theta^{Q^{\prime }}=\min_{\theta^{Q}}\frac{1}{N^{\prime }}\sum_{i=1}^{N}{\left(y_{i}-Q\left({\vec{s}}_{i},{\vec{a}}_{i}|\theta^{Q}\right)\right)}^{2}. \end{array}$$

The state transition tuples $\left\{{\vec{s}}_{i},\;{\vec{a}}_{i},\;R_{i}\;{\vec{s}}_{i+1}\right\}$, i=1,2,⋯,N¡, is directly extracted from the memory buffer. In terms of this case, $R_{t}\left({\vec{s}}_{t},{\vec{a}}_{t}\right)$ is defined as instantaneous energy efficiency for the UAV, denoted by

(14) $$\begin{array}{c} R_{t}\left(s_{t},a_{t}\right)=\frac{\sum_{i=1}^{M}B\left(T-\tau_{wp}\right)\log_{2}\left(1+\frac{P_{S}g_{i}^{2}\tau_{wp}}{\left(P_{at}g_{at}+\sigma^{2}\right)\left(T-\tau_{wp}\right)}\right)}{(P_{S}\tau_{wp}+P_{C}T)H}. \end{array}$$

Applying chain rule based gradient ascent, through extracting $M^{\prime }$ tuples in random from the memory buffer, we further calculate the renewed parameter set of actor network as follows

(15) $$\begin{array}{c} \theta^{\mu^{\prime }}=\theta^{\mu }+\frac{1}{M^{\prime }}\Sigma_{i=1}^{M^{\prime }}{▽}_{a}Q\left(s,a|\theta^{Q^{\prime }}\right){{|}_{s=s_{i},\;a=u\left(s_{i}\right)}{▽}_{\theta^{\mu }}\mu \left(s|\theta^{\mu }\right)|}_{s=s_{i}}. \end{array}$$

Then, we arrive at the updated parameter set of critic network as follows

(16) $$\begin{array}{c} \theta^{Q}=k_{1}\theta^{Q^{\prime }}+\left(1-k_{1}\right)\theta^{Q}, \end{array}$$

and the updated parameter set of actor network as follows

(17) $$\begin{array}{c} \theta^{\mu }=k_{1}\theta^{\mu^{\prime }}+\left(1-k_{1}\right)\theta^{\mu }, \end{array}$$

where $k_{1}$ is the exploration constant during the process of learning. The pseudo code is shown in Algorithm 1.

For each training iteration, the above mentioned training process needs to be repeated. After sufficient amount of training iterations, one actor network can be well trained to determine near-optimal transmission parameters in real time.
**Algorithm 1** pseudo-code for action policy trainingInitialize the critic network $Q(\vec{s},\vec{a}|\theta^{Q})$ and the actor network $\mu (\vec{s},\vec{a}|\theta^{\mu })$.Initialize the network parameter $\theta^{Q}$ and $\theta^{\mu }$.**for** episode ∈[1,2,⋯,M] **do**   Initialize a Rayleigh fading random process and generate initial state ${\vec{s}}_{t}$.   **for** t ∈[1,2,⋯,T] **do**     Select action ${\vec{a}}_{t}=\mu \left({\vec{s}}_{t}|\theta^{\mu^{\prime }}\right)+N_{t}$.     Randomly generate new state ${\vec{s}}_{t+1}$.     Use ${\vec{a}}_{t}$ and ${\vec{s}}_{t+1}$ to calculate the resulting reward $R_{t}$.     Save state transition tuple {${\vec{s}}_{t}$, ${\vec{a}}_{t}$, $R_{t}$, ${\vec{s}}_{t+1}$} into the memory buffer.     Extract a random minibatch of *N* state transition tuples {${\vec{s}}_{i}$, ${\vec{a}}_{i}$, $R_{i}$, ${\vec{s}}_{i+1}$} from the memory buffer, i=1,2,⋯,N.     Set expected value function $y_{i}$ to $r_{i}+\gamma Q\left({\vec{s}}_{i+1},\;\mu^{\prime }\left({\vec{s}}_{i+1}|\theta^{\mu^{\prime }}\right)|\theta^{Q^{\prime }}\right)$.     Update critic network parameter to $\theta^{Q^{\prime }}$ by minimizing the loss function as Equation (13).     Update actor network parameter by performing gradient ascent, shown as $\theta^{\mu^{\prime }}=\theta^{\mu }+{▽}_{\vec{a}}Q\left(\vec{s},\vec{a}|\theta^{Q^{\prime }}\right){|}_{\vec{s}={\vec{s}}_{i},\;\vec{a}=u\left({\vec{s}}_{i}\right)}{▽}_{\theta^{\mu }}\mu \left(\vec{s}|\theta^{\mu }\right)$.     Update corresponding network parameters as $\theta^{Q}=k_{1}\theta^{Q^{\prime }}+\left(1-k_{1}\right)\theta^{Q}$ and $\theta^{\mu }=k_{1}\theta^{\mu^{\prime }}+\left(1-k_{1}\right)\theta^{\mu }$.   **end for****end for**

## 4. Numerical Results

To test the validity of theoretical analysis and proposed optimization approach, we presented some numerical examples on important parameters, such as maximum energy efficiency, effective throughput. All the parameters, used in simulation, have been given in Table 1. To simplify the simulation process, $P_{at}$ is set to a very small value. All channel gains are assumed to be the same and follow rice distribution, all sensors’ positions are assumed to follow normal distribution in a circle area, and the UAV is assumed to be in stationary hovering state. Since the noise at the sensor is usually very tiny, Noise power consumption is set to be $1\times 10^{-8}$ Watt.

**Figure 3:**
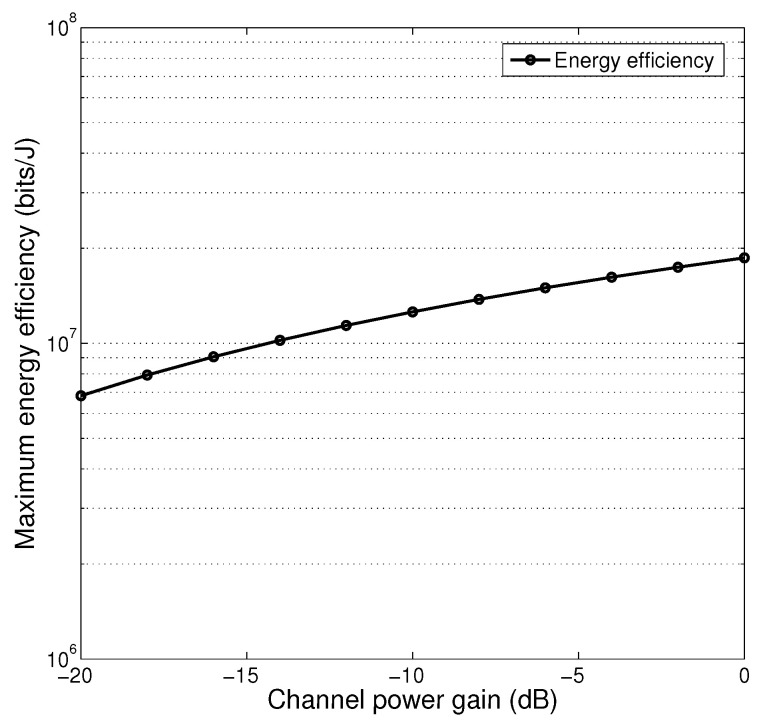
Miximum energy efficiency, TDMA mode.

Figure 3 presents the minimum energy consumption with respect to channel power gain under TDMA mode. Note that under TDMA mode, minimum energy consumption can be calculated by substituting optimal charging power and optimal charging duration into the objective function in Section 3.1. As expected by intuition, we see that energy efficiency monotonically increases with channel power gain. This is because that when channel condition deteriorate, charging power level needs to be higher for keeping the amount of collected data unchanged.

**Figure 4:**
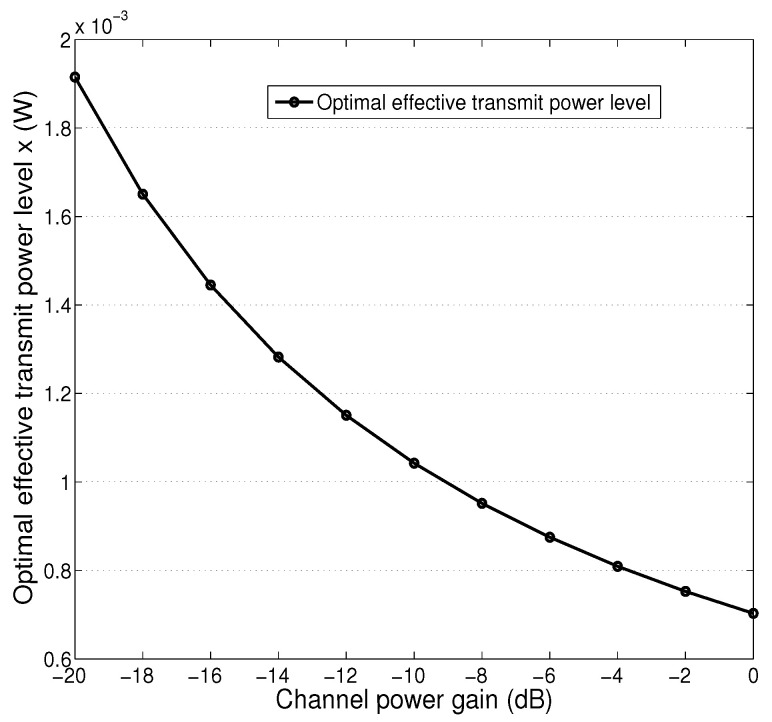
Optimal effective transmit power *x* with respect to channel power gain, TDMA mode.

Figure 4 presents the effective transmit power *x* with respect to channel power gain under TDMA mode. As expected, we see that the optimal power level generally decreases as channel power gain increases. We also see that descending velocity slightly decreases with the increasing channel power gain. This is because that when channel condition becomes better, less charging power is needed in general for collecting a fixed amount of data.

**Figure 5:**
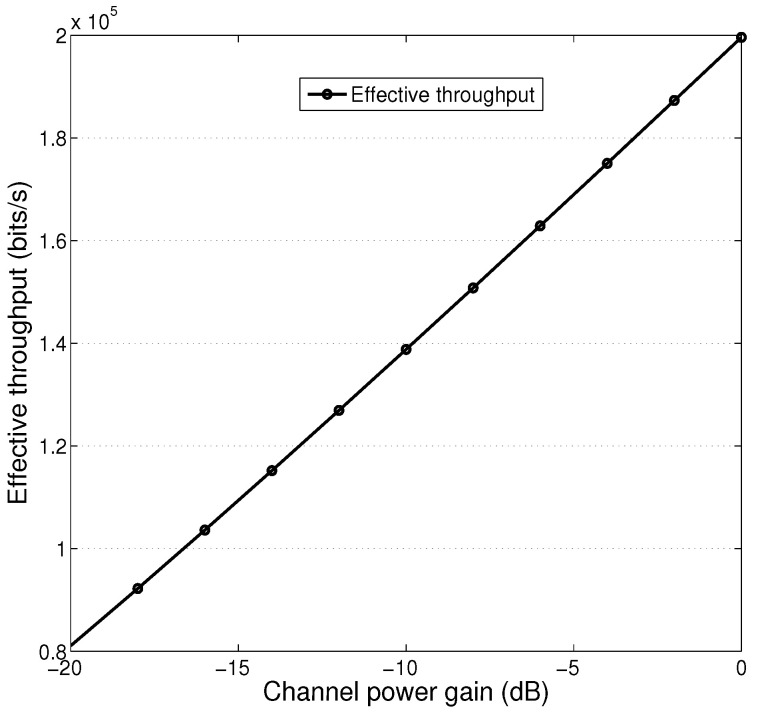
Effective throughput with respect to channel power gain, TDMA mode.

Figure 5 presents the effective throughput with respect to channel power gain under TDMA mode. We can see that the resulting throughput increases as channel power gain increases. This is because that received SNR increases as channel power gain increases. Note that since optimal charging power and charging duration varies with channel power gain, effective throughput increases with channel power gain in approximately linear manner.

**Figure 6:**
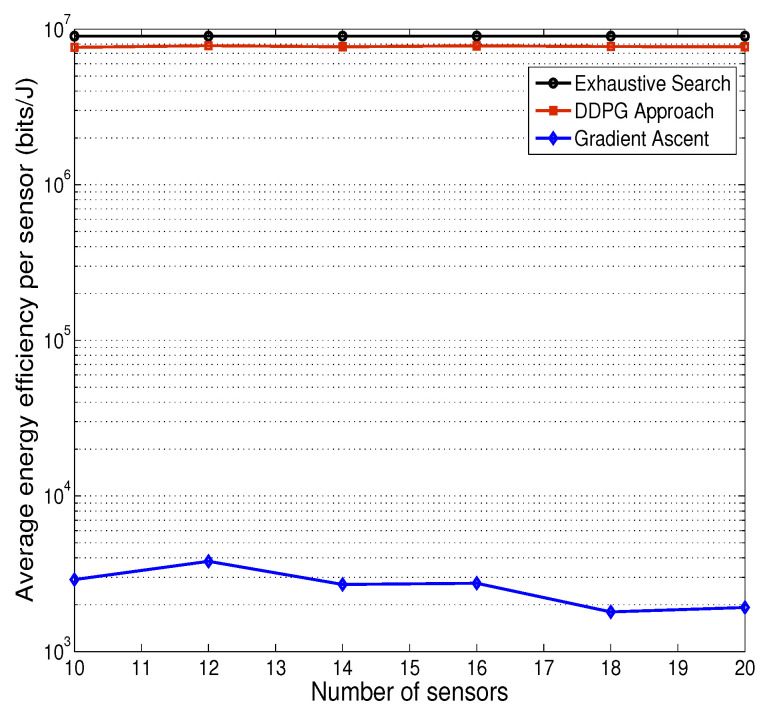
Effective energy efficiency per sensor, CDMA mode.

Figure 6 presents the numerical results on average energy efficiency per sensor, under CDMA mode, from gradient ascent, DDPG based approach and exhaustive search. It shows that the result from exhaustive search result is very close to that from DDPG approach and as such, our proposed DDPG approach achieves near-optimal performance. Furthermore, we compare the performance of DDPG approach with that of gradient ascent and show that DDPG approach has much better performance. Note that as the step size is relatively big, the result from gradient ascent is not good.

## 5. Conclusions

In this paper, we investigated the issue of energy efficiency maximization in wirelessly-powered sensor network with an attacker. In the case of TDMA, we maximized the energy efficiency through deriving closed-form optimal charging power, charging duration and transmission durations. Additionally, in the case of CDMA, we proposed a DDPG based approach to arrive at an action policy for determining near-optimal transmission parameters in real time, and reduce the calculation complexity. These results will be very valuable for arriving at energy efficient wirelessly-powered sensor network.

## Figures and Tables

**Table 1:** Parameters used in the simulation.

Parameter	Meaning	Value
$\sigma^{2}$	Noise Power	1$\;\times \;10^{-8}$ Watt
B	Available Bandwidth	10 kHz
ρ	Path Loss Exponent	2
P_*C*_	Circuit Power of Source Node	0.01 Watt
η	DC Conversion Efficiency	0.8

## Data Availability

Data are contained within the article.
